# Investigating sedentariness and health status of primary school teachers in Ghana

**DOI:** 10.1186/s12913-023-09925-3

**Published:** 2023-09-12

**Authors:** Edward W. Ansah, Mawuli Adabla, Norgbedzie Jerry, Eric A. Aloko, John E. Hagan

**Affiliations:** 1 Department of Health, Physical Education and Recreation (HPER), University of Cape Coast, Cape Coast, Ghana; 2 Neurocognition and Action-Biomechanics-Research Group, Faculty of Psychology and Sport Sciences, Bielefeld University, Postfach 10 01 31, Bielefeld, 33501 Germany

**Keywords:** Anxiety, Depression, Discomfort, Pain, Physical inactivity, Sedentariness, Self-rated health status

## Abstract

**Objective:**

Physical inactivity is linked to chronic illnesses and disabilities among workers, especially those in high demanding jobs like teachers. Despite the global prominence of sedentary behavior research, studies drawing the relationships between physical inactivity and multimorbidity among working teacher populations in low-and middle-countries remain untapped. This study assessed the sedentariness and health status of primary school teachers in Cape Coast Metropolis in Ghana.

**Method:**

This cross-sectional survey employed 1109 primary school teachers from the Cape Coast Metropolis in the Central Region of Ghana, targeting the entire population.

**Results:**

Generally, the teachers were highly sedentary and reported poor health status. Other results showed no difference in sedentariness across gender, (n = 1107, t= -0.32, p > 0.05). However, female teachers suffer more pain and discomfort, (n = 1103.51), t = − 3.07, p < 0.05), anxiety and depression, (n = 1099.56), t = − 2.85, p < 0.000), and poor health status (n = 1107), t = 2.14, p < 0.05), than their male counterparts. Also, pain and discomfort, anxiety and depression, health status and years of work significantly predicted sedentariness among the teachers, F (4, 1104) = 5.966, p = 0.00, R = 0.145, R^2^ = 0.029, adjusted R^2^ = 0.018.

**Conclusion:**

The findings suggest that individualized or personalized interventions are urgently needed to promote regular physical activity to improve the health status and reduce associated complications on the health and well-being, especially among the female primary school teachers. Given the health risks of sedentary lifestyle, behavioral interventions at the person-level (i.e., individualized- routine weekly physical activity programs) and built environmental restructuring (e.g., creation of walkways to encourage regular walking) could be done to improve physical activity behavior among teachers within the Cape Coast Metropolis, and perhaps beyond.

## What is already known about this subject?

For many individuals, employment can be a hindrance to regular physical activity engagement. Teachers are one group of workers with high level of sedentary behavior. Increased physical activity is protective against many chronic illnesses.

## What are the findings?

The teachers were highly sedentary and reported poor health status, but they did not differ in sedentariness across gender. Female teachers suffer more pain and discomfort, anxiety and depression, and poor health status compare with their male counterparts. Sedentariness among these teachers can be determined by perceived level of pain and discomfort, anxiety and depression, health status and years of working experience.

## Introduction

Work is limiting the activeness of many individuals, increasing their act of sedentariness and its associated health conditions [1]. Over the past few decades, sedentary lifestyle has evolved as an important global public health concern. For instance, in the general population, sedentary behavior has been associated with an increased risk of cardiovascular conditions, mood disorders, and all-cause mortality [2]. Globally, each year about 3.2 million deaths are attributed to sedentary behavior, making sedentariness the fourth leading cause of death globally [3]. Sedentary behavior is any waking behavior that is characterized by energy expenditure of less than 1.5 metabolic equivalents of task (MET) while in a sitting, reclining or lying position [4].

Many individuals spend more of their wake hours at sitting positions. For instance, globally, 55% of people (23% male and 32% female), aged 18 + years, recorded very insufficient physical activity participation [5]. In addition, evidence indication that one in every five adults in Europe takes little or no physical activity, and higher levels of physical inactivity was found among eastern European countries [6]. In the European Union (EU), two thirds of the adult population do not reach recommended levels of physical activity [7]. As a result, physical inactivity is estimated to deprive Europeans over 8 million days of healthy life every year [7].

Not all persons experience the effects of sedentariness at the same level. In sub-Saharan Africa, physical inactivity-related non-communicable diseases (NCDs) are responsible for about 3 million deaths annually [8]. These death rates are expected to increase to 80% in the near future if urgent actions are not taken [9]. In Ghana, 94,400 (43%) of the total deaths in 2016 were attributed to NCDs that are related to sedentary behavior [6]. In as much as physical activity participation is known to confer numerous health benefits, most people are still not making conscious effort to stay physically active [10]. Thus, regular physical activity is rapidly on the decline in all populations. Such declines are attributable to the various factors. Magnon, Vallet, and Auxiette [11] suggest that sedentary behavior is associated with employment, and that, employment is an important determinant of sedentariness [12].

The burden of disease caused by physical inactivity does not only affect the victims, their families and friends, but it also put a huge strain on health care systems, leading to sickness absenteeism and presenteeism and associated loss of productivity [13]. Thus, many categories of workers may suffer physical inactivity due to several work factors including, stress, unavailability of exercise facilities and work pressures. Therefore, workers with higher workload are more likely to be sedentary.

The teaching profession is one of the jobs that can be very stressful due to work overload. The demands of teaching (e.g., work overload), and associated poor working conditions have consistently been reported in low- and middle-income countries. Such work climate predisposes teachers to occupational diseases such as high blood pressure, gastrointestinal problems, and musculoskeletal disorders which negatively impact teachers’ health and well-being [14, 15]. Furthermore, at the psychological level, physical in-activeness aggravates the levels of stress, anxiety, and general fatigue [16]. Unfortunately, participation of teachers in physical activity is said to be very less [5, 16], even though regular exercise activity has numerous health benefits [17]. For instance, a study [18] on insufficient free-time physical activity and occupational factors in Brazilian public-school teachers showed a prevalence of 71.9% physical inactivity, which resulted in poor work output. The authors reiterated that the adverse working conditions were increasing the prevalence of sedentariness among the teachers. However, the authors failed to explore how insufficient physical activity affected the health of teachers, since health and well-being contribute to quality teaching, training and production of quality graduates [19], for community and national development. A similar study [20] on the effects of physical activity on perceived general health of teachers in Spain showed that physical activities at low levels were more prevalent among the teachers (39%). In addition, it was revealed that teachers recorded moderate (26%) and high (35%) levels of physical activity. Thus, majority of teachers in Spain do not meet the WHO recommendation of 150 min of moderate-to-vigorous intensity physical activity per week [21]. Though this study provides interesting findings, it centered on the three categories of physical activity as identified by the International Physical Activity Questionnaire (IPAQ) including, walking, moderate and vigorous levels, and failed to explore the relationship between sitting time (sedentary) and health status of the teachers [22]. Therefore, teachers are likely to become sedentary individuals which in turn compromises their health and well-being.

Teachers at the basic schools in Ghana are classroom teachers who are employed by the Ghana Education Service. These teachers are required to teach all the subjects in their respective classes. The teachers spend the better part of their day in the classroom teaching or marking assignments, exercises and attending to other student-related activities. Thus, the teaching duties create very little or no avenue for physical activity. Meanwhile, rapid urbanization has brought significant variations in modifiable lifestyle, such as sudden shift and access to public transport, more hours of usage of digital technology, including social media which have significantly increased sedentariness [23, 24]. Observation indicates that teachers are frequently using their smartphones, laptops, and other electronic gadgets browsing social media content of interest during break times [25]. Unfortunately, the built environment in most African context especially Ghana and Cape Coast Metropolis are not exercise friendly [26]. This attitude contributes to sedentary behavior of teachers as most of them travel to school and other places by vehicles or motorcycles for most days of the week [27].

Sedentariness may predispose many primary school teachers in Cape Coast to NCDs. However, studies on physical inactivity or sedentariness and their health implications in academics have always focused on students [28]. There seems to be a dearth of empirical information, particularly on sedentariness among teachers in the African region, which is a major hinderance for appropriate policy and personalized interventions aimed at reducing NCD burden. Thus, there is the need to focus research on physical inactivity and its adverse effects on the health status of teachers in Ghana. Therefore, the purpose of this study was to analyse the relationship among sitting time and anxiety/depression, sitting time and pain/discomfort and sitting time and the general health status as well as gender difference by sitting time among primary school teachers in the Cape Coast Metropolis of Ghana.

## Materials and methods

### Participants’ selection

A cross-sectional descriptive survey was employed to study 1109 primary school teachers from the Cape Coast Metropolis. Though, all the 1142 (census) primary school teachers in the Cape Coast Metropolis were invited to take part in the study.

### Study instruments

Two pre-existing instruments (IPAQ short form and EQ-5D-3 L) were used to collect data from the participants [29]. The IPAQ comprises four generic items that measure health-related physical activity. The items focus on the number of days and times a person spent in the last seven (7) days doing vigorous, moderate activities, walking, and sitting or lying down. This instrument recorded high values of validity and reliability coefficients when used across Europe (0.82 and 0.81), Africa (0.79 and 0.84), Asia (0.77 and 0.79) and America (0.86 and 0.82), respectively [30]. According to Group T. E [31]., the EQ-5D-3 L consists of two parts, EQ-5D descriptive system and EQ visual analogue scale (EQ VAS). The EQ-5D-3 L descriptive system comprises five dimensions: mobility, self-care, usual activities, pain/discomfort and anxiety/depression. Each dimension has three levels: no problems, some problems, extreme problems. The EQ VAS records the respondent’s self-rated health on a vertical, visual analogue scale. The endpoints are labelled ‘Best imaginable health state’ and ‘Worst imaginable health state’ (the instrument, response options and ratings can be found here; https://euroqol.org/publications/user-guides). This instrument has been used and validated across nations in Africa, 0.88 and 0.79, 0.77 and 0.81, for mobility, self-care, usual activities, pain/discomfort and anxiety/depression, respectively [32]. Data from our pilot study also yielded a reliability and validity of 0.85 and 0.84 for VAS and 0.70 and 0.81 for Eq. 5D-3 L, respectively.

### Procedure

Ethical clearance was granted by the University of Cape Coast’s Institutional Review Board (ID-UCCIRB/CES/2019/30). Also, permissions were sought from the Regional and Metropolitan Directorates of Education of Ghana Education Service, and the heads of the various basic schools in the Metropolis. The participants were contacted at their schools through the heads in the schools and the questionnaire was distributed to the teachers. We engaged and trained research assistants who helped in the data collection. The purpose of the study, issues of confidentiality, anonymity and voluntary participation in the study were carefully written at the introductory page of the questionnaire which each participant was expected to read and understand before taking part in the study. Study participants were asked to sign an consent form prior to answer the questionnaire. Participants were given two days to complete and return the questionnaire, because the researchers did not want the study to disrupt instructional time of the teachers. This data was collected between August and December, 2019, where each participant spent about 20 min responding to the instrument.

### Data analysis

Data were entered into Statistical Package for Social Science, IBM, version 21 for windows and went through a thorough screening process before the analysis. The differences in gender by health status, pain and discomfort, anxiety and depression and sedentariness were analysed using independent sampled t-test. Also, multiple linear regression was computed to predict sedentariness from health status, pain and discomfort, anxiety and depression and years of teaching experience of the teachers.

## Results

Out of the total population of 1142 teachers in the Metropolis, 1109 (about 97% response rate) took part in this survey. These were teachers who were present at their schools at the time of data collection. They comprised 556 males and 553 females, from kindergarten = 171, primary school = 434 and Junior High School = 504.

The age of the teachers ranged between 20 and 45 years, thus, a youthful cohort. Majority of the teachers in this study area are trained teachers with a minimum qualification of diploma certificate. Others hold higher qualifications such as bachelor’s degree and master’s degree. Their teaching experience ranged from one to 25 years. However, a few of the teachers have taught for less than one (1) year.

To determine the difference in sedentariness by gender, pain and discomfort, anxiety and depression, and health status, four separate independent t-tests were calculated. The test results showed no statistically significant difference in sedentariness based on gender, (n = 1107, t= -0.32, p > 0.05), and that sedentariness among males (M = 32.26, SD = 61.53) did not differ from females (M = 33.46, SD = 64.90). Levene’s test indicated equal variance, (F = 0.037, p = 0.85). However, there was statistically significant difference in self-reported health status by gender, (n = 1107, t = 2.14, p < 0.05), with male teachers (M = 52.50, SD = 36.63) recording an improved self-reported health than their female colleagues (M = 47.88, SD = 35.43). Levene’s test indicated equal variance (F = 3.36, p = 0.067). Other results show statistically significant difference in pain and discomfort by gender, (n = 1103.51, t = − 3.07, p < 0.05), with the males (M = 1.23, SD = 0.48) reporting less pain and discomfort compared to the females teachers (M = 1.33, SD = 0.51). Furthermore, there was statistically significant difference in anxiety and depression by gender, (t = 1099.56, t = − 2.85, p < 0.000), with females (M = 1.3, SD = 0.51) reporting slightly higher levels of anxiety and depression scores than the male teachers (M = 1.21, SD = 0.47).

Furthermore, multiple linear regression was computed to predict sedentariness of the teachers using pain and discomfort, anxiety and depression, health status, and years of teaching experience. The test results showed that the model involving all the four independent variables significantly predicted sedentariness of the teachers, F (4, 1104) = 5.966, p = 0.00, R = 0.145, R^2^ = 0.029, adjusted R = 0.018. Thus, pain and discomfort, β = 0.176, (n = 1105, t = 4.40, p < 0.05) and anxiety and depression, β = 0.102, t (1105) = -2.58, p < 0.05 significantly predicted sedentariness among the teachers. Furthermore, years of teaching experience significantly predicted sedentariness, β = 0.077, (n = 1105, t= -2.49, p < 0.05), but not the health status of the teachers, β = 0.045, (n = 1105, t = 1.48, p > 0.05) (see Table 1 for details).

**Table 1:** Predictors of sedentariness of basic school teachers in Cape Coast

Variable	B	beta	T	P
**Constant**	23.140	.	3.606	0.000
**Pain and discomfort**	22.363	0.176	4.403	0.000*
**Anxiety and depression**	-13.089	0.102	-2.580	0.010*
**Years of Teaching experience**	− 0.724	0.077	-2.488	0.013*
**Health status**	0.078	0.045	1.484	0.138
**R**	0.145			
**R** ^**2**^	0.029			
**Adjusted R**	0.018			

F-ratio = 5.966, df = (4, 1104), p < 0.05

## Discussion

This study assessed sedentariness and associated health status of primary school teachers in the Cape Coast Metropolis in Ghana. The findings indicated that though sedentariness was high among the teachers, this did not significant differ by gender. This is likely because there are no gender specific roles in the teaching profession in Ghana. Teaching schedules, relative to time-tables, class periods, time allocations, and non-academic obligations such as sports and religious activities within each academic year are not gender-specific. Thus, teaching and teaching-related activities are similar across curricula schedules among male and female teachers in Ghana.

Occupationally, both men and women alike spend same amount of time in schools performing similar tasks [33]. These routines may involve sitting and standing with an energy expenditure of less than 1.5 METs per day leading to high level of sedentary behavior [34], even though studies have suggested that men are generally active than women [35]. Badr, Rao and Manee [33] explained that, there is no gender difference in sedentary life among teachers because both men and women are equally likely to have jobs that involve sitting for long hours. Also, there are similar pressures on both genders to keep up with their work responsibilities, which may limit such workers from engaging in regular physical activity.

The health and well-being implications of sedentariness are numerous. Sedentary behaviors associated with teaching such as long hours of sitting to mark assignments, mark attendance, attending meetings, writing lesson notes, among others, predispose teachers (both male and female alike) to cardiovascular diseases and other non-communicable diseases. Since standing and sitting are daily routines for all teachers in the basic schools [36], it is not surprising that teachers in this study do not differ in sedentary behavior. However, it is worrying to know that basic school teachers in Cape Coast Metropolis spend about 16 h (8 h at work and 8 h sleeping) out of the 24 h inactive. Unfortunately, sedentariness predisposes teachers to NCDs, the fourth leading cause of all deaths globally [37]. We believe that such sedentariness-induced ill health condition would negatively impact quality teaching in the schools [37].

The finding further showed that female teachers reported poorer self-rated health status. Gender difference in health status among the teachers can vary greatly [38]. Usually, women tend to spend more time in sedentary activities than men, and this can compromise their health and quality of work. Again, women are more likely to suffer from chronic diseases such as obesity, diabetes, and heart diseases due to their more sedentary lifestyles, that in turn compromise their ability to be more productive at work including teaching [39]. Also, women are more likely to suffer from depression, anxiety, and other mental health conditions due to complications of sedentary lifestyles [40]. Fortunately, higher levels of physical activity engagement can have a positive impact on male teachers’ health and teaching outputs [41]. This may provide some amount of protection to men from chronic illnesses and other health issues [42]. Overall, it is important for both genders to engage in regular physical activity to improve and maintain their health and well-being, contribute proactively to quality teaching and training of students in the country [43]. This becomes essential as our findings revealed that more females are teaching at the primary education level in the Metropolis.

The results further revealed that female teachers suffered and reported higher degree of pain and discomfort compared to their male counterparts. Gender differences in pain and discomfort perception among the teachers is an important area of research [44]. Recently, Prieto-González et al. [45] found that, female teachers tend to be more sedentary than male teachers, and this difference was associated with greater pain and discomfort perceived by the females. Earlier, Vaghela, and Parekh [46] also found that more female teachers reported higher levels of neck, shoulder, and lower back pain. Similar evidence [47] identified a link between sedentary behavior and musculoskeletal pain in teachers, with female teachers more likely to experience and report severe pain. Therefore, it is important to consider the role of gender in sedentary life and how that effect pain and discomfort among teachers.

The finding again indicated that female basic school teachers in Cape Coast Metropolis reported higher levels of anxiety and depression than their male counterparts. Anxiety and depression continue to be a problem among teachers [48], that adversely affect teachers’ physical health and mental well-being [49]. Moreover, it is noted that teaching is one of the professions that exposes workers to stress, depression and anxiety due to a combination and multifaceted characteristics of work-related activities [50]. For instance, the evidence is that female teachers are more likely to experience anxiety and depression than male teachers [51]. This gender gap is most pronounced when teachers are overwhelmed by their workloads and lost control over their work [52]. Baluyos, Rivera, and Baluyos [53] aslo found that female teachers tend to experience higher levels of job insecurity than male teachers, that may compromise the anxiety and depression levels of the female teachers. This may lead to a greater risk of burnout due to the levels of stress from work overload. Thus, female teachers need practical interventions (e.g., anxiety-stress reduction techniques) to improve their health, safety and well-being including mental health and be able to deliver quality teaching.

We found that levels of pain and discomfort, anxiety and depression, years of teaching experience, and health status predicted the level of sedentariness among the teachers. Despite all the variables significantly predicted sedentariness of the teachers, pain and discomfort contributed more to the model. This outcome presupposes that, the more sedentary these teachers are, the more pain and discomfort they are likely to feel and report. El-Tallawy et al. [54] espoused that, a sedentary lifestyle is associated with increased pain and discomfort. Thus, physical inactivity can lead to a number of disorders, including weakened muscles, joint stiffness, and poor posture [55] accumulating into pain. Additionally, prolonged sitting can strain the spine and increase the risk of back pain. The opposite can also be true, that pain and discomfort can lead to sedentariness of the teachers. Hence, regular physical activity can help to strengthen muscles, improve posture, and reduce the risk of developing chronic pain and discomfort [56].

### Limitations

Though this study highlighted sedentariness and associated health status of the primary school teachers in the Cape Coast Metropolis, it has some limitations. This is a survey that comes with self-reported data. Probably, adding interview would have given us a more in-depth data from the perspectives of the teachers. Also, about 371 of the teachers did not provide time duration for their physical activities per week. Thus, it was impossible to calculate their weekly physical activity MET and to determine their PA levels. Fortunately, they provided the time duration for their sitting time per week, which enabled us to calculate their level of sedentariness. It is important that another study explore how physical activity and sedentariness of teachers contribute to their quality teaching and students’ academic performance.

### Practical implications

Unfortunately, physical inactivity or sedentariness is increasing the burden of NCDs. Available evidence suggests that people, regardless of gender, who are insufficiently physically active have heightened risk of all-cause mortality, compared to those who engage in at least 30 min of moderate-intensity physical activity regularly [57]. Improving health and well-being of teachers are key to quality teaching and production of well-qualified graduates. Moreover, physical inactivity among the teachers may increase their propensity for overweight and obesity, and the rate of other chronic illnesses and disorders. Thus, not only the teachers, their families, students and the education system that suffer due to the ill health of these teachers, the health care system would be treating large numbers of complex NCDs. Thus, pragmatic efforts such as policy and other evidence-based health interventions are needed to improve physical activity levels of these teachers. The current findings suggest the importance of creating opportunities for regular physical activity to decrease the levels of sedentariness among the teachers. Creating personalized or individualized interventions could be helpful in improving energy imbalance among these teachers. This initiative can boost physical activity routines at work through health promotion programs. Moreover, environment restructuring (e.g., creating walkways and physical workspace design) that is user friendly could help increase physical activity behavior among these teachers. Future studies are required to investigate the clinical effectiveness of physical activity on mental health effects across a larger teacher population in Ghana.

## Conclusion

Primary school teachers in the Cape Coast Metropolis were highly sedentary, with the female teachers reporting poorer health status including, severe pain and discomfort, elevated anxiety and depression than the male teachers. It was also observed that sedentariness among these group of teachers was associated with pain and discomfort, anxiety and depression, health status and years of work of these teachers. The findings suggest that regular physical activity, a major health promoting behavior, is highly recommended for these teachers to aid in the prevention and management of potential NCDs among the teachers. Thus, tailored multi-faceted behavioral activities like light-to-moderate-to-vigorous intensity physical activity (e.g., walking, skipping), including playing sports and cycling are essential for these teachers. These activities would help improve the health status of these teachers, which would in turn, boost teaching productivity in the Cape Coast Metropolis.

## Data Availability

The datasets generated and/or analysed during the current study are available in the Open Science Framework, and it is here: https://osf.io/c4yz9/.
